# Supplementary material for: Comparative Analysis of Clinical and CT Findings in Patients with SARS-CoV-2 Original Strain, Delta and Omicron Variants
Source: Biomedicines. 2023 Mar 14;11(3):901. doi: 10.3390/biomedicines11030901 (PMC10046064; doi:10.3390/biomedicines11030901)
Supplement: Supplementary file 1 [file biomedicines-11-00901-s001.zip › biomedicines-2239077-supplementary.pdf]

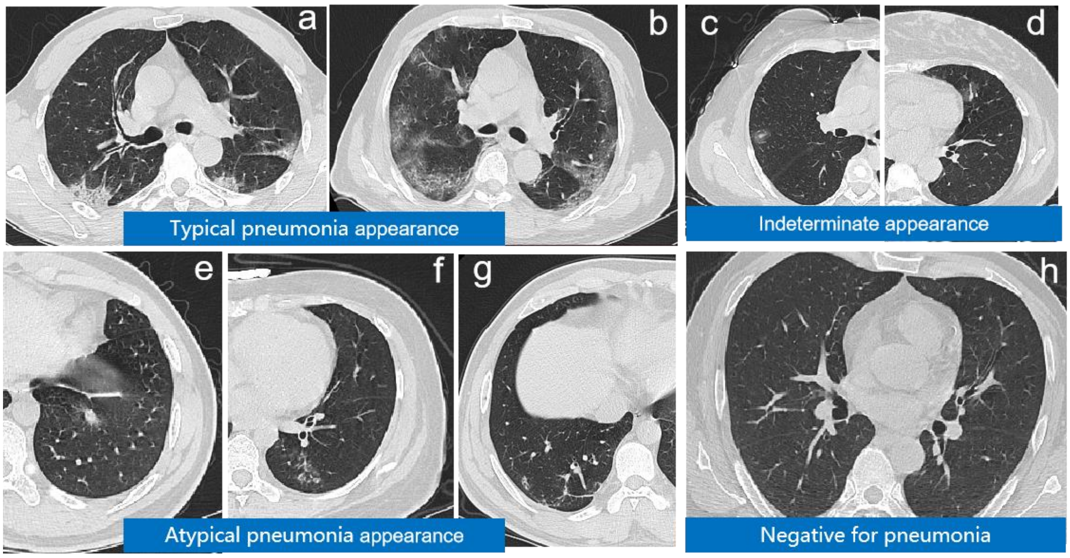

Figure S1. COVID-19 pneumonia imaging classification. (a, b) Typical CT imaging features for COVID-19: bilateral and peripheral GGO with superimposed interlobular septal thickening. (c, d) Indeterminate appearance for COVID-19: focal GGO lacking a specific distribution or nonrounded. (e-g) Atypical appearance, isolated lobar or segmental consolidation without GGO(e), Discrete small nodules (centrilobular, "tree-in bud") (f, g), (h) Negative for COVID-19 CT scan.

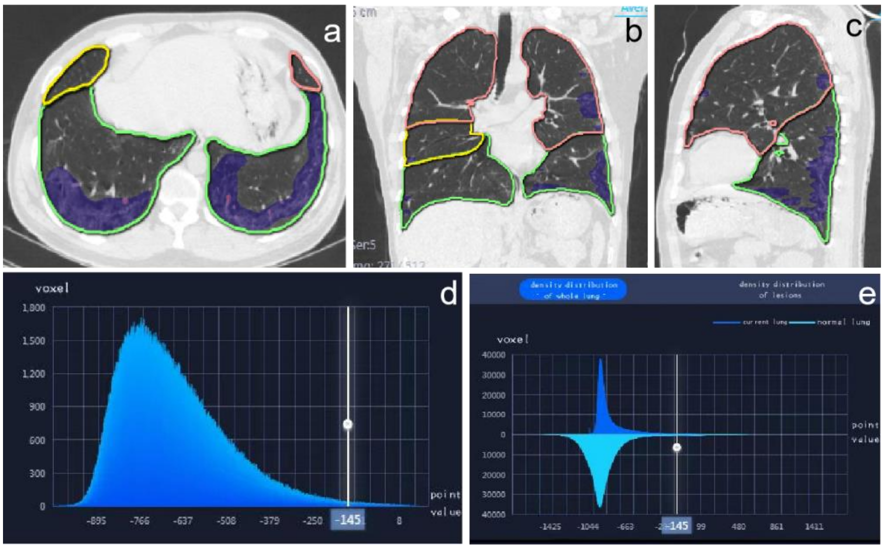

Figure S2. Quantitative CT analysis by artificial intelligence software.

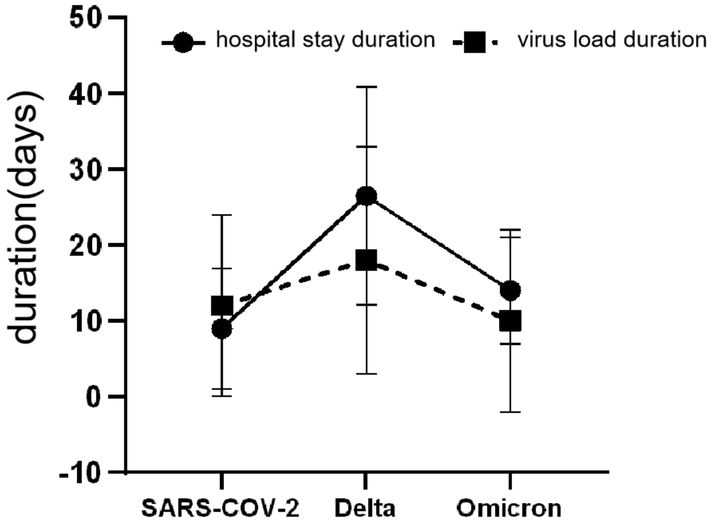

Figure S3. Duration of infection and hospital stays among three groups.

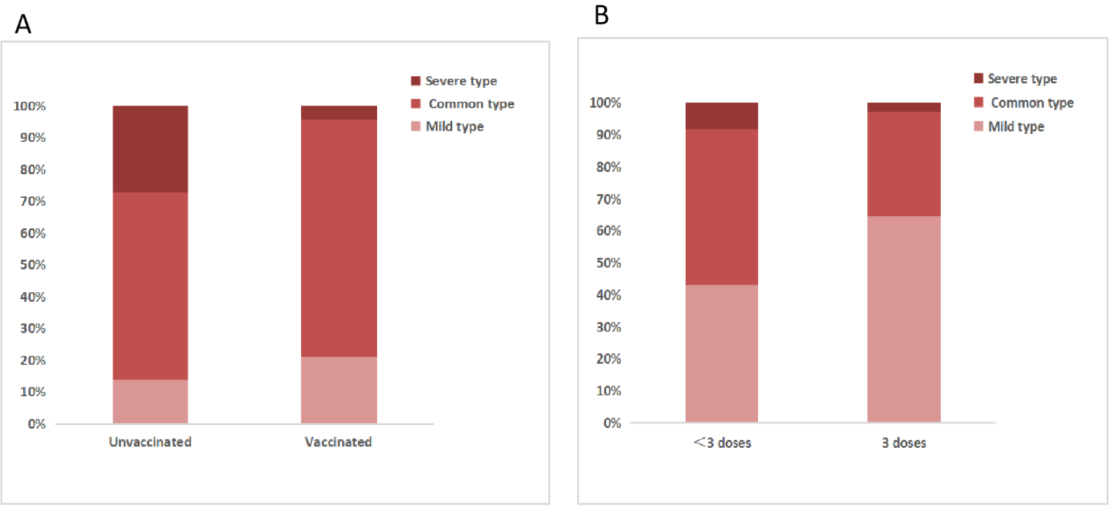

Figure S4. The proportion of patients with each category of clinical severity by variant or vaccine status. (A) Comparison of clinical severity in vaccinated versus unvaccinated patients infected with the Delta variant; (B) Comparison of clinical severity of Omicron variant infection in patients who received the third dose of vaccine versus those who did not receive the third dose

Table S1 The criteria of clinical severity

Clinical severity	Diagnostic criteria
mild	with mid clinical symptoms but without radiological findings of pneumonia
moderate	with fever, respiratory symptoms, etc., and radiological findings of pneumonia
severe	with any one of the following：<br>1. respiratory distress， respiratory rate (RR) ＞30 breaths per minute<br>2. oxygen saturation ≤93% at rest<br>3.ratio of partial pressure of arterial oxygen (PaO2) to fraction of inspired oxygen (FiO2) ≤300mmHg
critical	with any one of the following：<br>1. with respiratory failure and requiring mechanical ventilation；<br>2. developing shock<br>3. combined with other organ failure treated in the intensive care unit.

Table S2 Multivariable logistic regression analysis models for the association between clinical severity and listed variables

Clinical severity-moderate	Coefficient	SE	z value	Wald χ2	*P* value	OR value	OR value 95% CI
SARS-CoV-2 variants	-1.183	0.252	-4.698	22.071	0.000	0.306	0.187 ~ 0.502
Gender	0.051	0.287	0.178	0.032	0.859	1.052	0.600 ~ 1.845
Age	0.045	0.010	4.617	21.320	0.000	1.046	1.026 ~ 1.066
Comorbidities	0.367	0.372	0.987	0.973	0.324	1.444	0.696 ~ 2.994
Number of vaccination	-0.368	0.142	-2.593	6.726	0.010	0.692	0.524 ~ 0.914
Intercept	0.817	0.520	1.571	2.467	0.116	2.263	0.817 ~ 6.270
Clinical severity-severe	B	SE	z value	Wald χ2	*P* value	OR value	OR value 95% CI
SARS-CoV-2 variants	-1.553	0.337	-4.608	21.238	0.000	0.212	0.109 ~ 0.410
Gender	0.591	0.377	1.566	2.452	0.117	1.806	0.862 ~ 3.784
Age	0.076	0.013	5.684	32.310	0.000	1.079	1.051 ~ 1.107
Comorbidities	0.348	0.452	0.769	0.591	0.442	1.416	0.584 ~ 3.436
Number of vaccination	-0.607	0.248	-2.450	6.001	0.014	0.545	0.335 ~ 0.886
Intercept	-2.284	0.751	-3.041	9.251	0.002	0.102	0.023 ~ 0.444

McFadden $R2$=0.265

Cox & Snell $R2$=0.395

Nagelkerke $R2$=0.464

Table S3 Comparison of laboratory examinations of different strains

Laboratory examination tests	Normal Range	Original strain (n=245)	Delta variant (n=90)	Omicron variant (n=168)	P value
Duration of positive nucleic acid	-	12(8,20)	18(11-26)	10(1-13)	<0.001
ORF	negative	--	23.6(11.3-29.8)[c]	29.9(24.8-33.2)	<0.001
N gene	negative	--	22.6(14.1-28.3)[c]	27.6(24.6-32)	<0.001
IgM	0	--	0(0-0)[c]	0.07(0.05-0.18)	<0.001
IgG	0	--	0(0-0)[c]	22.1(2.1-149.6)	<0.001
Leukocyte count ($\times10^9$/L)	3.5-9.5	6.7(5.1-10.7) [a,b]	5.8(4.6-7.1)	6.3(4.8-8.0)	0.372
Lymphocyte count ($\times10^9$/L)	1.1-3.2	0.8(0.51-1.2) [a,b]	1.2(0.8-1.8)	1.2(0.8-1.8)	<0.001
Hemoglobin (g/L)	130.0-175.0	113(104-125) [a,b]	148 (130.5-155)[c]	141(27-149)	0.457
D-dimer (μg/L)	<0.5	2.7(0.7-11.1) [a,b]	0.3(0.2-0.6)	0.25(0.14-0.39)	<0.001
Glucose(mmol/L)	3.9-6.1	8.1(6.5-10.3) [a,b]	6.2(4.9,6.7)	6.2(5.3-7.0)	<0.001
ALT (U/L)	5.0-50.0	55(26-86.3) [a,b]	22(13-38)	29(24.3-42.8)	0.181
AST (U/L)	15.0-40.0	43(28-66) [a,b]	28(25-39)	21(17.0-28.0)	0.828
LDH (U/L)	109.0-245.0	343(263-500) [a,b]	200(162.5-271.3)[c]	156 (138.5-170)	0.277
IL-6 (pg/mL)	<7.0	9.5(7.0-12.8)	10.6(7.4-15.0)[c]	7.9(4.9-11.6)	0.013
Procalcitonin (ng/mL)	<0.5	0.05(0.05-0.12) [a,b]	0.02(0.02-0.05)[c]	0.013(0.002-0.028)	0.996
CRP(mg/L)	<8.0	46.3(12.8-112.2) [a,b]	7.3(0.6-22.3)	3.7(1.2-10.6)	<0.001

The data are presented as mean value standard deviation or medians (interquartile ranges).

ALT：alanine aminotransferase；AST：aspartate transaminase； LDH：lactate dehydrogenase； IL-6：interleukin-6；ESR: erythrocyte sedimentation rate；CRP：C-reactive protein

a, $p < 0.05$ between Original strain and Delta strain；

b, $p < 0.05$ between Original strain and Omicron strain；

c, $p < 0.05$ between Delta strain and Omicron strain.

Table S4 Univariable logistic regression analysis models for the association between total CT score and listed variables

Variable	Coefficient	SE	z value	Wald χ2	*P* value	OR value	OR value 95% CI
SARS-CoV-2 variants	-0.956	0.208	-4.602	21.178	0.000	0.384	0.256 ~ 0.578
Gender	0.299	0.256	1.169	1.366	0.243	1.349	0.817 ~ 2.228
Age	0.005	0.008	0.662	0.439	0.508	1.005	0.989 ~ 1.022
Comorbidities	0.491	0.298	1.647	2.712	0.100	1.634	0.911 ~ 2.931
Number of vaccination	-0.025	0.154	-0.165	0.027	0.869	0.975	0.722 ~ 1.318
Intercept	1.054	0.467	2.259	5.103	0.024	2.870	1.150 ~ 7.163

Total CT score cutoff ＞5

McFadden *R2*= 0.138

Cox & Snell *R2*=0.116

Nagelkerke *R2*=0.196

Table S5 AI evaluation results of pneumonia with different SARS-CoV-2 strains

CT characteristics	Original strain (n=245)	Delta variant (n=90)	Omicron variant (n=168)	P value
Lesion volume				
Bilateral lungs	255(64,578) [a,b]	60.5(31.9,112)	45.8(27.6,103)	< 0.001
right lung	157(28.8,367) [a,b]	39.9(13.8,85.1)	29.8(7.5,40.3)	
left lung	83(21,248.6) [a,b]	32.9(0,59.6)	26.1(0,62.6)	0.001
CT value of lesions				
Bilateral lungs	312(84,639) [a,b]	66(36,100)	53.3(27.6,93.7)	<0.001
right lung	201(44,392) [a,b]	41(24,80.7)	37(41.6,69.7)	<0.001
left lung	122(30,251) [a,b]	25(14.2,50.6)	22.0(10.8,57)	<0.001
CT value of whole lung	1.8(1.5,2.1) [a,b]	2.1(1.5,2.4)	2.2(1.8,2.6)	<0.001
Whole lung volume				
Bilateral lungs	3847±980	4022±1031	4525±1245	0.062
right lung	2060±535	2125±510	2391±640	0.260
left lung	1788±469 [b]	1897±534	2134±619	0.046
Proportion of lesions				
Bilateral lungs	6.6(1.7,18.8) [a,b]	2.6(2.1,7.5)	1.2(0.8,3.9)	<0.001
right lung	6.7(1.3,19.2) [a,b]	1.1(0.2,5.1)	0.9(0.2,2.8)	<0.001
left lung	5.4(1.1,18.1) [a,b]	0.15(0,3.1)	0.2(0,5.4)	<0.001

a, $p < 0.05$ between Original strain and Delta strain;

b, $p < 0.05$ between Original strain and Omicron strain;

c, $p < 0.05$ between Delta strain and Omicron strain.

Table S6 Multivariable logistic regression analysis models for the association between clinical severity and listed variables in patients infected with Omicron variant

Clinical severity-moderate	Coefficient	SE	z value	Wald $\chi 2$	*P* value	OR value	OR value 95% CI
Vaccination booster	-1.16	0.442	-2.625	6.891	0.009	0.313	0.132 ~ 0.745
Gender	-0.27	0.426	-0.634	0.402	0.526	0.763	0.331 ~ 1.759
Age	0.087	0.017	5.086	25.869	0	1.091	1.055 ~ 1.129
Comorbidities	-0.122	0.573	-0.213	0.045	0.832	0.885	0.288 ~ 2.720

Table S6 Multivariable logistic regression analysis models for the association between clinical severity and listed variables in patients infected with Omicron variant

Intercept	-3.287	0.766	-4.293	18.433	0	0.037	0.008 ~ 0.168
Clinical severity-severe	Coefficient	SE	z value	Wald χ2	*P* value	OR value	OR 值 95% CI
Vaccination booster	-2.088	1.027	-2.034	4.135	0.042	0.124	0.017 ~ 0.927
Gender	0.732	1.006	0.727	0.529	0.467	2.078	0.289 ~ 14.934
Age	0.1	0.035	2.833	8.028	0.005	1.105	1.031 ~ 1.184
Comorbidities	18.741	5296.981	0.004	0	0.997	137805126.2	0.000 ~ Infinity
Intercept	-24.724	5296.981	-0.005	0	0.996	0	0.000 ~ Infinity

McFadden *R2*=0.329

Cox & Snell *R2*=0.423

Nagelkerke *R2*=0.521

Table S7 Multivariable logistic regression analysis models for the association between lung involvement and listed variables in patients infected with Omicron variant

Variable	Coefficient	SE	z value	Wald χ2	*P* value	OR value	OR value 95% CI
Vaccination booster	-0.782	0.337	-2.319	5.378	0.020	0.458	0.236 ~ 0.886
Gender	-0.367	0.333	-1.103	1.217	0.270	0.693	0.361 ~ 1.330
Age	0.013	0.012	1.085	1.177	0.278	1.013	0.990 ~ 1.036
Comorbidities	0.081	0.497	0.162	0.026	0.871	1.084	0.409 ~ 2.870
Intercept	-0.140	0.559	-0.251	0.063	0.802	0.869	0.290 ~ 2.601

McFadden *R2*= 0.051

Cox & Snell *R2*=0.067

Nagelkerke *R2*=0.090
